# Influence of body mass index on the reliability and validity of ultrasound assessments in adolescent idiopathic scoliosis: An original research

**DOI:** 10.1371/journal.pone.0335472

**Published:** 2025-11-24

**Authors:** JoJo Yiying Zou, Shirley Lok Yi Ho, Babak Hassan Beygi, Changliang Luo, Man Sang Wong

**Affiliations:** 1 Department of Biomedical Engineering, The Hong Kong Polytechnic University, Hong Kong SAR; 2 School of Rehabilitation, Kunming Medical University, Kunming, China; Iran University of Medical Sciences, IRAN, ISLAMIC REPUBLIC OF

## Abstract

**Objectives:**

Clinical ultrasound provides a non-invasive method to assess spinal curvature in adolescent idiopathic scoliosis (AIS). However, the reliability and validity of ultrasound assessment may be affected by body mass index (BMI). This study investigated the impact of BMI on the reliability and validity of ultrasound assessments in AIS.

**Design:**

165 participants with suspected AIS were recruited for both ultrasound and radiographic assessments. Lateral spinal curvature was measured on ultrasound imaging using the spinous process method and on X-ray using the Cobb method. The same operator performed two ultrasound scans for each participant, and two independent raters measured the images (Rater 1: 1^st^ and 2^nd^ scans; Rater 2: 1^st^ scan only). Intra-operator and inter-rater reliabilities were assessed using intraclass correlation coefficients (ICCs), and validity was assessed by correlating ultrasound angles with Cobb angles using Pearson’s r. Participants were categorized by BMI tertiles and BMI-for-age percentiles for subgroup analyses.

**Results:**

The second BMI tertile (16.2–18.5 kg/m^2^) exhibited the highest reliability: ICC (2,1)=0.83 (95% CI: 0.73–0.90) intra-operator and 0.88 (95% CI: 0.76–0.94) inter-rater. By BMI-for-age classification, the normal-weight group demonstrated the highest reliability (ICC = 0.78 and 0.84) and the lowest standard error of measurement (2.6° and 2.3°). Validity was strongest in the 18.1–24.4 kg/m^2^ BMI group (r = 0.85), compared with 12.3–15.8 kg/m^2^ (r = 0.58) and 16.0–17.7 kg/m^2^ (r = 0.61).

**Conclusions:**

The reliability of ultrasound assessments of spinal curvature was highest in adolescents in the second BMI tertile and in the normal-weight group, while underweight and overweight groups showed lower reliability. Correlation with Cobb angles was strongest in the 18.1–24.4 kg/m² BMI group (r = 0.85), suggesting ultrasound performs best when soft-tissue conditions are neither minimal nor excessive. These findings suggest that BMI should be considered when interpreting ultrasound measurements and when designing screening protocols for AIS.

## Introduction

Adolescent idiopathic scoliosis (AIS) is a three-dimensional spinal deformity that affects approximately 2–3% of adolescents [1]. It is typically diagnosed and monitored using radiographic imaging, with the Cobb angle serving as the gold standard for quantifying the degree of scoliosis curvature [2]. Regular follow-up is essential during puberty and until skeletal maturity to monitor curve progression and evaluate the effectiveness of interventions such as physiotherapy or orthotic management [1]. However, the need for frequent radiographic evaluations raises concerns about cumulative radiation exposure, which has been linked to long-term health risks, including an increased risk of breast cancer in adolescents [3]. These risks highlight the importance of exploring alternative assessment methods that minimize radiation exposure.

Given these concerns, clinical ultrasound imaging has gained attention for scoliosis assessment, offering real-time visualization of spinal structures without ionizing radiation [4]. In ultrasound assessments, spinal deformity can be estimated by analyzing the midline curve formed by spinous process (SP) landmarks and calculating the angle based on the turning points of the two most tilted vertebrae [5]. Previous studies have demonstrated strong correlations between spinal curvature measurements from ultrasound imaging and Cobb angles measured from radiography, with reported mean absolute difference (MAD) ranging from 2.5° ± 1.9° to 4.9° ± 3.2° [5,6]. Additionally, the SP angle measured from ultrasound imaging has shown high intra- and inter-rater reliability, with intraclass correlation coefficients (ICCs) ranging from 0.88 to 0.99 for intra-rater reliability and 0.84 to 0.95 for inter-rater reliability [5,7,8]. These findings suggest that ultrasound could serve as a non-ionizing modality for scoliosis monitoring, particularly in scenarios requiring frequent evaluations.

Despite its potential, the quality and accuracy of ultrasound imaging could be influenced by body composition, which is often approximated using body mass index (BMI) as a widely used indicator [9]. Excessive soft tissues, such as subcutaneous fat, can attenuate ultrasound waves and reduce image resolution and penetration depth [10], while insufficient soft tissue may hinder probe contact and acoustic coupling, compromising image quality [11]. Studies have shown that increased fat thickness obscures anatomical landmarks and reduces measurement accuracy, as seen in abdominal ultrasound, where imaging quality is often inversely correlated with BMI [12]. Surveys of sonographers similarly indicate that patients with normal BMI generally yield clearer ultrasound images compared to those with higher BMI [13]. In spinal ultrasound, one study reported weaker correlations between ultrasound and radiographic measurements in the normal-weight and overweight groups compared to the underweight group among children and adolescents [14], while another study found no significant differences in validity across BMI categories for patients aged 8–40 years [7]. These inconsistencies may arise from variations in ultrasound systems, population characteristics, and BMI classification criteria. Many spinal ultrasound studies have excluded participants with higher BMIs (e.g., > 23 or >25 kg/m²) [7,8,15], yet the effects of BMI on intra-operator and inter-rater reliability remain unclear.

Therefore, the study aimed to investigate the influence of BMI on the reliability and validity of spinal ultrasound imaging in scoliosis assessment for adolescents. The intra-operator and inter-rater reliability of spinal curvature angles, measured based on the alignment of SP in ultrasound images, was assessed, along with their correlation with radiographic Cobb angles across different BMI groups. It was hypothesized that individuals within the normal-weight category, which represents a mid-range BMI, would exhibit the highest reliability and validity in spinal ultrasound assessments. The findings may inform the clinical applicability of ultrasound as a non-invasive tool for scoliosis monitoring.

## Materials and methods

### Study design and participants

This observational cross-sectional study utilized data collected from a large community service project that provided scoliosis screenings for adolescents. Initial screenings were conducted in schools and involved Adam’s forward bending test and scoliometer measurements [16]. Adolescents with a trunk rotation exceeding 5 degrees, which is considered indicative of possible scoliosis [16], were referred to a spine assessment center for further evaluation. At the center, participants underwent spinal ultrasound imaging and standard radiographic examination to confirm the presence and severity of scoliosis and assess the feasibility of ultrasound assessment in clinical settings. Participants were included in this study if they: 1) had complete records of age, sex, body weight, and height; 2) were between 10 and 18 years old; and 3) had completed both ultrasound and radiographic assessments. The exclusion criteria were: 1) scoliosis secondary to syndromic, neuromuscular, or congenital causes; 2) leg-length discrepancies greater than 1 cm; or 3) prior surgical treatment. This study was approved by the local Institutional Review Board (HSEARS20221012006), and informed consent was obtained from all participants and their parents or guardians. Data were collected from 31^st^ March 2023–31^st^ December 2024, and data collection and management strictly adhered to the approved research protocol.

BMI was calculated as weight in kilograms divided by height in meters squared (kg/m²). Two methods were employed to classify BMI: (1) a tertile-based classification that divided participants into first, second, and third tertile BMI groups to ensure balanced group sizes; and (2) the CDC BMI-for-age classification (https://www.cdc.gov/bmi/child-teen-calculator/bmi-categories.html) (underweight: < 5^th^ percentile; normal: 5^th^–85^th^ percentile; overweight: > 85^th^ percentile) for consistency with established health standards [17].

### Scoliosis assessment

An upright whole-spine X-ray was performed as a routine diagnostic procedure, with the participant standing with arms on their sides [1]. Ultrasound assessments were conducted in the same posture using the Scolioscan system (Model SCN801, Aitrasound Medical Imaging Ltd, Hong Kong). All ultrasound scans followed a standardized protocol, with probe placement guided by anatomical landmarks [8]. The probe was initially positioned slightly below the L5 vertebra and moved upward along the spinous processes to slightly above T1 vertebra in a continuous motion, while maintaining consistent skin contact [8]. To enhance posture stability during scanning, a rigid frame with four supporting points (aligned with the clavicle anterior concavities and bilateral anterior superior iliac spines) was used [8]. All scans were conducted using predefined acquisition parameters: frequency 7.5 MHz, imaging depth 7.1 cm, focus depth 3.6 units, contrast 118 dB, brightness 34 dB, and default time gain compensation (TGC) settings. Each participant underwent two scans, both performed by the same certified operator. Each scan lasted approximately 30 seconds to keep a stable posture and obtain reliable images, ensuring consistent image acquisition. The second scan was conducted 30–60 seconds after the first, allowing time for system adjustments (e.g., software interaction, reapplying ultrasound gel). Participants maintained their general posture between scans but were allowed to make posture adjustments for comfort during the interval.

**Fig 1:**
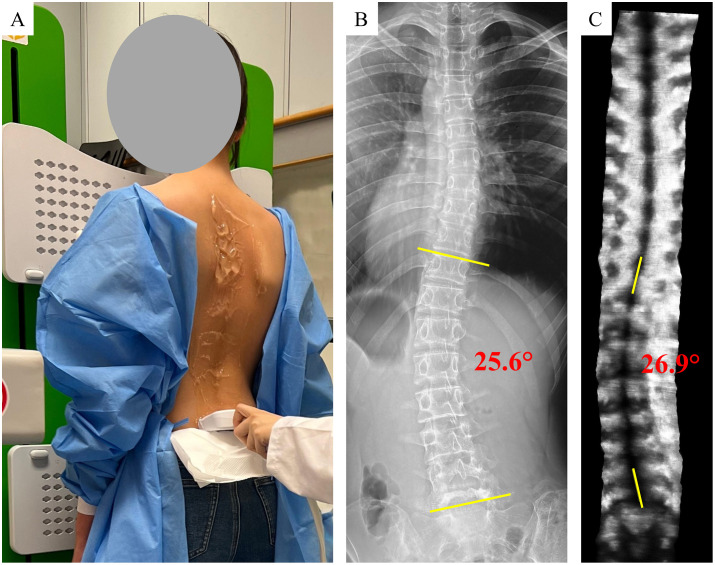
Scoliosis assessment in this study: A) ultrasound scan using the Scolioscan® system; B) standard posteroanterior radiograph showing a Cobb angle of 25.6°; C) corresponding coronal ultrasound image showing a curvature angle of 26.9° using SP method.

The Cobb angle from radiographs was measured as the angle between the superior endplate of the most tilted upper vertebra and the inferior endplate of the most tilted lower vertebra [2]. The ultrasound angle (US angle) was determined by identifying the two most tilted vertebrae in the scoliotic curve, manually drawing two short lines at their turning points on the coronal image with reference to the SP, and the angle between them was automatically calculated based on their orientations [8]. Only major spinal curvatures were analyzed in both radiographic and ultrasound assessments. To ensure measurement consistency, two licensed rehabilitation therapists completed standardized training [8]. Rater 1, with three years of spinal ultrasound experience, measured curvatures from both ultrasound scans to evaluate the intra-operator reliability. The rater was blinded to the previous results to minimize memory and recall bias. Measurements from Rater 1 and Rater 2, who was newly trained, on the first ultrasound scan were compared to evaluate inter-rater reliability. Both raters were blinded to participants’ identities and each other’s measurements. A third rater with three years of clinical experience measured the Cobb angles from the radiographs, and all measurements were verified by a certified orthopedic surgeon. Cobb angle measurements were conducted first to identify the location and magnitude of major spinal curvatures, followed by US angle measurements, which were performed without knowledge of the specific vertebral levels. All measurements were performed manually using medical imaging software (MicroDicom Viewer for Windows, Bulgaria). No automated or semi-automated methods were used. **Fig 1** illustrated the scoliosis assessment process in the study.

### Statistical analysis

The statistical analyses were performed using IBM SPSS software (version 27.0). Descriptive statistics summarized participant characteristics (age, sex, BMI) and scoliosis curvatures from radiograph and ultrasound imaging. Data normality was evaluated using the Shapiro-Wilk test. Differences in US and Cobb angles among BMI subgroups were analyzed using one-way analysis of variance (ANOVA). If normality assumptions were violated, the Kruskal-Wallis test was used as a non-parametric alternative. Intra-operator and inter-rater reliability of US angle measurements were assessed using intraclass correlation coefficients (ICCs) with a two-way random effects model and absolute agreement. ICC (2,1) values were interpreted using Currier criteria: < 0.60 questionable, 0.60–0.79 moderate, ≥ 0.80 very reliable [18]. Measurement precision was estimated using the standard error of measurement (SEM), with lower SEM values indicating higher measurement precision. The reliability of Cobb angle measurements has been previously reported, with ICC values ranging from 0.96 to 0.98 for both intra- and inter-rater assessments [19]; therefore, it was not re-evaluated in this study. The validity of the US angle was assessed by comparing the average angle measured by Rater 1 from the two US scans with the Cobb angles, with both assessments conducted on the same day. The MAD quantified measurement differences, while Pearson correlation coefficients (r) evaluated their relationship (0.20–0.40 weak, 0.40–0.60 moderate, 0.60–0.80 strong, 0.80–1.00 very strong) [20]. Agreement between ultrasound and radiographic measurements was further assessed using Bland-Altman analysis, which evaluated systematic bias and limits of agreement (LoA).

## Results

**Fig 2:**
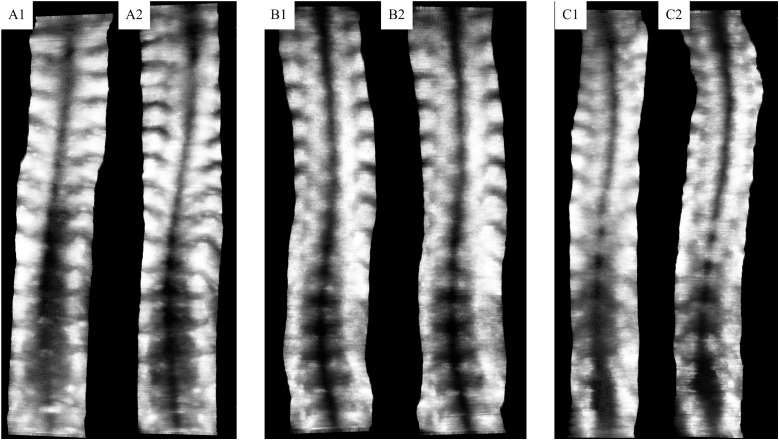
Spinal ultrasound images of subjects (A&B&C) with different BMIs across two scans (1&2). A: underweight (BMI 13.7 kg/m^2^); B: Normal-weight (BMI 17.4 kg/m^2^); C: Overweight (BMI 24.1 kg/m^2^).

A total of 253 adolescents from the screening program underwent full spine radiography for further diagnostic evaluation. Among them, 165 met the initial inclusion criteria and were included in the primary analysis. Considering that significant vertebral rotation may affect the accuracy of spinal ultrasound assessments [8], a subgroup of 116 participants with apical vertebral rotation (AVR) less than 5°, as measured by the Perdriolle method [21], was analyzed separately. Among all eligible participants, 72 (BMI range: 12.3–24.4 kg/m^2^) completed both assessments on the same day, and their data were used for validation analysis. Representative ultrasound images across different BMI levels are shown in **Fig 2**.

### Reliability of ultrasound assessment

**Table 1** shows the intra-operator and inter-rater reliability of US angle assessments across different BMI groups. No significant differences in US angles were observed among the three BMI groups using either the tertile-based classification or the CDC BMI-for-age classification. In the tertile-based BMI classification, the second tertile BMI group demonstrated the highest intra-operator reliability (ICC = 0.83, 95% CI: 0.73–0.90) and smallest SEM (2.3°), followed by the third tertile BMI group (ICC = 0.81, SEM = 2.9°), both indicating high reliability. The first tertile BMI group showed moderate reliability (ICC = 0.67, 95% CI: 0.50–0.79) and the largest SEM (3.2°). In terms of inter-rater reliability, the second tertile BMI group again showed the highest reliability (ICC = 0.88, 95% CI: 0.76–0.94) and smallest SEM (2.1°), followed closely by the third tertile BMI group (ICC = 0.79), and the first tertile BMI group had the lowest ICC (0.66). Using the CDC BMI-for-age percentile classification, similar patterns were observed. The normal-weight group demonstrated moderate intra-operator reliability (ICC = 0.78, 95% CI: 0.71–0.84) and high inter-rater reliability (ICC = 0.84). In contrast, both the underweight and overweight groups showed wide 95% confidence intervals (0.50–0.87 and 0.36–0.90, respectively). For inter-rater reliability, the underweight and overweight groups exhibited lower ICC (0.65 and 0.63), consistent with findings from the tertile-based classification. These trends remained consistent in the subgroup (n = 116), as detailed in the table in **S1 Table**.

**Table 1:** Intra-operator and inter-rater reliability of ultrasound assessments across different BMI groups (n = 165).

	Age (yrs) Mean ± SD	Sex (Female/Male)	BMI (kg/m^2^) Range Mean ± SD	US angle (°) Mean ± SD			Intra-operator reliability (1^st^ scan vs. 2^nd^ scan)		Inter-rater reliability (Rater 1 vs. Rater 2)	
				1^st^ scan (Rater 1)	2^nd^ scan (Rater 1)	1^st^ scan (Rater 2)	SEM (°)	ICC (2,1) [95% CI]	SEM (°)	ICC (2,1) [95% CI]
**Tertile-based classification**										
First Tertile BMI (n = 56)	11.9 ± 1.8	39/17	12.3–16.3 15.1 ± 0.8	14.9 ± 6.0	14.0 ± 6.2	12.0 ± 5.6	3.2	0.67 [0.50, 0.79]	3.2	0.66 [0.32, 0.82]
Second Tertile BMI (n = 53)	13.3 ± 1.7	41/12	16.4–18.6 17.4 ± 0.7	14.1 ± 5.9	13.3 ± 5.8	12.8 ± 6.2	2.3	0.83 [0.73, 0.90]	2.1	0.88 [0.76, 0.94]
Third Tertile BMI (n = 56)	13.1 ± 1.5	32/24	18.8–30.7 21.2 ± 2.2	14.1 ± 6.9	13.2 ± 7.1	11.5 ± 6.5	2.9	0.81 [0.69, 0.88]	3.0	0.79 [0.48, 0.90]
Group comparison (p)	<0.001	0.075	<0.001	0.820	0.646	0.375	n.a.	n.a.	n.a.	n.a.
**CDC BMI-for-Age Percentile Classification**										
Underweight (n = 26)	12.9 ± 1.9	12/14	12.3–16.5 14.7 ± 1.0	16.0 ± 6.4	14.8 ± 7.4	12.4 ± 7.1	3.3	0.74 [0.50, 0.87]	3.7	0.65 [0.21, 0.85]
Normal-weight (n = 124)	12.8 ± 1.8	94/30	14.5–23.1 17.9 ± 2.0	13.7 ± 6.0	13.0 ± 6.0	12.0 ± 5.8	2.6	0.78 [0.71, 0.84]	2.3	0.84 [0.67, 0.91]
Overweight (n = 15)	12.3 ± 1.6	6/9	21.0–30.7 23.8 ± 2.3	17.0 ± 6.9	15.3 ± 7.3	12.5 ± 6.9	3.5	0.72 [0.36, 0.90]	3.9	0.63 [0.03, 0.88]
Group comparison (p)	0.666	<0.001	<0.001	0.053	0.302	0.972	n.a.	n.a.	n.a.	n.a.

SEM: standard error of measurement; ICC: intraclass correlation coefficient; CI: confidence interval; n.a.: not applicable.

### Validity of ultrasound assessments

The participants who completed both assessments on the same day were categorized into BMI tertile groups within the validation cohort: first tertile (V1), second tertile (V2), and third tertile (V3). **Table 2** shows the validation results using the Cobb angle as the reference standard. The MAD between the US and Cobb angles was largest in the V1 group (8.4° ± 6.6°), indicating lower agreement between the two modalities. In contrast, the V2 and V3 groups exhibited smaller MAD values (7.4 ± 6.0° and 6.2 ± 4.1°, respectively), suggesting better agreement. Pearson correlation analysis revealed a very strong positive correlation in the V3 group (r = 0.85, 95% CI: 0.68–0.93), a strong correlation in the V2 group (r = 0.61, 95% CI: 0.28–0.82), and a moderate correlation in the V1 group (r = 0.58, 95% CI: 0.22–0.79). When using the CDC BMI-for-age classification, further subgroup comparisons were not conducted due to the small sample size in the individuals with overweight status (n = 6), which limits the reliability of statistical analysis.

**Table 2:** Validation of ultrasound assessments across BMI groups using same-day ultrasound and radiography.

	Sex (Female/Male)	Age (yrs) Mean ± SD	BMI (kg/m^2^) Range	US angle (°) Mean ± SD	Cobb angle (°) Mean ± SD	MAD (°) Mean ± SD	Pearson’s r [95% CI]
**Tertile-based classification**							
V1 (n = 24)	17/7	11.6 ± 1.9	12.3–15.8	15.4 ± 6.7	20.2 ± 11.8	8.4 ± 6.6	0.58 [0.22, 0.79]
V2 (n = 24)	18/6	13.3 ± 1.8	16.0–17.7	13.6 ± 6.4	19.6 ± 9.4	7.4 ± 6.0	0.61 [0.28, 0.82]
V3 (n = 24)	17/7	12.8 ± 1.5	18.1–24.4	14.4 ± 6.1	19.8 ± 9.2	6.2 ± 4.1	0.85 [0.68, 0.93]
Group comparison (p)	0.934	0.005	<0.001	0.625	0.923	0.678	n.a.

V1: validation cohort, first BMI tertile; V2: validation cohort, second BMI tertile; V3: validation cohort, third BMI tertile; MAD: mean absolute difference; CI: confidence interval; n.a.: not applicable.

**Fig 3:**
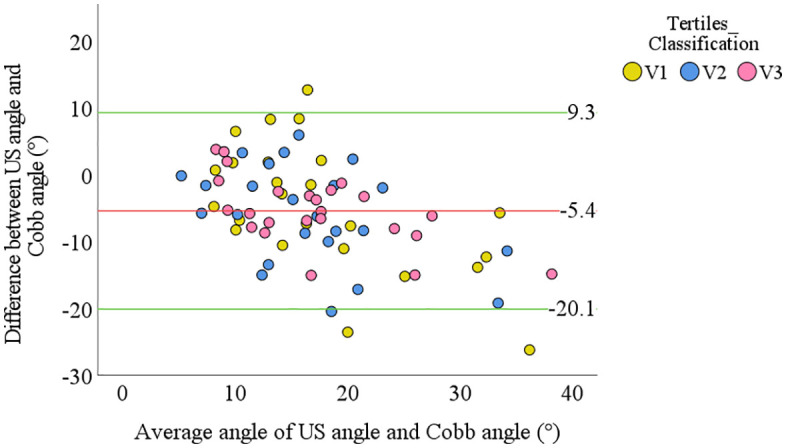
Bland-Altman analysis of US and Cobb angles across BMI tertiles. V1: validation cohort, first BMI tertile; V2: validation cohort, second BMI tertile; V3: validation cohort, third BMI tertile.

The Bland-Altman plot further illustrates the agreement between US and Cobb angles across different BMI groups (**Fig 3**). The mean difference was 5.4°, indicating an underestimation by the US angle, with LoA ranging from −20.1° to 9.3°. The V1 group exhibited greater variability, with several data points exceeding the LoA, suggesting lower measurement consistency. In contrast, the V2 and V3 groups showed better agreement, as their data points were more tightly clustered within the LoA.

## Discussion

The study investigated the influence of BMI on the reliability and validity of ultrasound assessments of spinal curvature, using the Cobb angles as the reference standard. The findings show that BMI affects both intra-operator and inter-rater reliability. Specifically, individuals in the lowest and highest BMI tertiles (the first and the third) exhibited greater measurement variability, while those in the middle tertile (the second) showed the highest reliability and smallest measurement error. In the validation analysis, participants with a BMI of 18.1–24.4 kg/m^2^ showed the strongest agreement between the US and Cobb angles.

Among participants in the third tertile, excessive soft tissue thickness reduced image clarity and obscured bony landmarks, especially in the lumbar region (**Fig 2**, panels C1 and C2), as previously reported [7,14]. The SP line often appeared broader and less defined, making it more challenging to determine its orientation. These challenges are particularly relevant for less experienced raters, who may find it difficult to distinguish the true spinal deformity.

Conversely, the first tertile was also associated with lower intra-operator and inter-rater reliability, as well as reduced validity when compared to radiographic measurements. Two main factors may contribute to this observation. First, prominent bony structures, such as the SP and winged scapula, can make probe stabilization more difficult and prevent full contact with the skin during scanning [14,22]. This may lead to image shifting and artifacts, potentially resulting in misinterpretation of spinal curvature and greater measurement variability. As shown in **Fig 2**, panels A1 and A2, although bony landmarks were clearly visible, the spinal landmarks in the lumbar and upper thoracic regions were not fully captured. Robotic scanning systems may be particularly affected, as the prominence of SPs can hinder automated stabilization [15,22,23]. Second, suboptimal ultrasound settings, such as excessive gain or inadequate TGC, may further compromise image quality in individuals with less soft tissue. For individuals in the first tertile, parameter adjustments such as increasing transducer frequency, reducing scanning depth, and optimizing gain may enhance the resolution of superficial anatomical structures and improve landmark visualization [14]. Additionally, reducing probe pressure or using aids like silicone pads may enhance stability and reduce imaging shifting.

In contrast, the second BMI tertile likely benefits from an optimal balance, where soft tissue provides sufficient probe stabilization and good contact with the skin without obscuring key anatomical landmarks. Similar trends were observed using both the tertile BMI classification and the CDC BMI-for-age categories. In both cases, the middle BMI group demonstrated higher measurement reliability and narrower confidence intervals than the corresponding lower and higher BMI groups. These consistent patterns, including those from the subgroup (AVR < 5°, see table in **S1 Table**), support a strong association between BMI and ultrasound measurement reliability. Due to clinical workflow constraints, default scanning settings were applied across all participants to ensure procedural efficiency. However, this approach may have limited the accuracy of assessments in participants with atypical body composition. These findings highlight the potential benefits of tailoring ultrasound protocols to individual body composition. It is worth noting that the V3 group in this study (18.1–24.4 kg/m^2^) primarily represented individuals within the normal to slightly overweight range, with only three subjects classified as overweight in clinical standards. This suggests that the scanning parameters used were most compatible with this BMI range. Future studies should explore optimized ultrasound settings for different BMI categories to improve the accuracy and reliability of scoliosis assessments.

In this study, BMI was used as a proxy for body composition due to its practicality and feasibility in a large-scale scoliosis screening. While BMI does not directly measure body fat and cannot distinguish between fat mass, lean mass, or variations in bone density and fat distribution, it remains a widely used and accepted tool in both clinical and research settings. Alternative methods, such as dual-energy X-ray absorptiometry (DEXA) [24], bioelectrical impedance analysis (BIA) [25], and skinfold thickness measurement (plicometry) [26], were not included in this study due to ethical concerns and practical limitations: DEXA involves additional radiation exposure, plicometry requires skilled operators and is prone to inter-rater reliability [27], and BIA results can be influenced by factors such as hydration status [28]. Despite its limitations, BMI has demonstrated strong correlations with percentage body fat (PBF) (r = 0.735–0.799) and fat mass index (FMI) (up to r = 0.944) in large-scale studies with over 18,000 participants [9]. It can also explain 58–66% of the variance in PBF in adolescents and adults, supporting its utility as an accessible estimate of body composition in population-based research [29].

To further support the use of BMI as a proxy in this study, a supplementary analysis was conducted using an independent dataset of 144 local adolescents. In this sample, fat mass measured by BIA showed a strong overall correlation with BMI (r = 0.77), with even higher correlations observed in the underweight (r = 0.89) and overweight (r = 0.88) subgroups, and a moderate correlation in the normal-weight group (r = 0.67). Although not part of the main study, these findings provide additional support for the use of BMI as a practical surrogate for body composition in this population. Future studies should consider using combined methods, such as BIA and plicometry, to gain a more comprehensive understanding of how body composition affects spinal ultrasound measurements in AIS.

This study also has other limitations. In the validity analysis, the narrow BMI range (12.3–24.4 kg/m^2^) limits generalizability to individuals with higher BMI. Expanding the sample size, particularly for underweight and overweight individuals, could provide better insights into BMI’s impact on ultrasound assessment accuracy. Additionally, this study used SP measurements but did not compare reliability across different anatomical landmarks (e.g., transverse processes [5,30] and the center of the lamina [14,31]). Future research should explore whether alternative landmark-based measurements improve reliability.

## Conclusions

This study demonstrated that BMI influences the reliability and validity of ultrasound in assessing AIS, with the highest reliability observed in the second BMI tertile and the normal-weight group, and generally lower reliability in the underweight and overweight groups. The correlation with Cobb angles was strongest in the 18.1–24.4 kg/m² group (r = 0.85), suggesting that ultrasound performs best when soft-tissue conditions are neither minimal nor excessive. Therefore, BMI should be considered when interpreting ultrasound assessments and designing AIS screening protocols, as well as during the long-term follow-up of AIS treatment.

## Supporting information

S1 Table Intra-operator and inter-rater reliability of ultrasound measurements across different BMI groups (n = 116). (DOCX)
