# Supplementary material for: Influence of body mass index on the reliability and validity of ultrasound assessments in adolescent idiopathic scoliosis: An original research
Source: PLoS One. 2025 Nov 24;20(11):e0335472. doi: 10.1371/journal.pone.0335472 (PMC12643305; doi:10.1371/journal.pone.0335472)
Supplement: S1 Table — (DOCX) [file pone.0335472.s001.docx]

**S1 Table** Intra-operator and inter-rater reliability of ultrasound assessments across different BMI groups (n =116).

|  | Age  (yrs)  Mean ± SD | Sex  (Female/Male) | BMI  (kg/m^2^)  Range  Mean ± SD | US angle (°)  Mean ± SD | | | Intra-operator reliability  (1^st^ scan vs. 2^nd^ scan) | | Inter-rater reliability  (Rater 1 vs. Rater 2) | |
| --- | --- | --- | --- | --- | --- | --- | --- | --- | --- | --- |
|  |  |  |  | 1^st^ scan  (Rater 1) | 2^nd^ scan  (Rater 1) | 1^st^ scan  (Rater 2) | SEM (°) | ICC (2,1)  [95% CI] | SEM (°) | ICC (2,1)  [95% CI] |
| **Tertile-based classification** | | | | | | | | | | |
| First Tertile BMI (n = 36) | 11.4 ± 1.3 | 24/12 | 12.3 – 16.0  15.0 ± 0.8 | 14.8 ± 4.7 | 13.2 ± 5.9 | 11.6 ± 5.1 | 3.2 | 0.53  [0.25, 0.73] | 2.9 | 0.59  [0.13, 0.81] |
| Second Tertile BMI (n = 39) | 13.2 ± 1.6 | 30/9 | 16.4 – 18.5  17.2 ± 0.7 | 12.6 ± 5.2 | 12.2 ± 5.3 | 11.2 ± 5.6 | 2.4 | 0.77  [0.60, 0.87] | 2.0 | 0.85  [0.68, 0.93] |
| Third Tertile BMI (n = 41) | 13.0 ± 1.5 | 24/17 | 18.6 – 30.7  21.0 ± 2.3 | 12.5 ± 6.1 | 11.1 ± 5.1 | 10.5 ± 5.6 | 2.9 | 0.69  [0.49, 0.82] | 2.4 | 0.83  [0.55, 0.82] |
| Group comparison (p) | <0.001 | 0.218 | <0.001 | 0.138 | 0.368 | 0.545 | n.a. | n.a. | n.a. | n.a. |
| **CDC BMI-for-Age Percentile Classification** | | | | | | | | | | |
| Underweight  (n = 18) | 12.6 ± 1.9 | 7/11 | 12.3 – 16.5  14.7 ± 1.0 | 14.3 ± 4.9 | 13.3 ± 7.2 | 10.5 ± 5.6 | 3.1 | 0.70  [0.37, 0.88] | 3.2 | 0.55  [0.003, 0.82] |
| Normal-weight  (n = 87) | 12.7 ± 1.6 | 66/21 | 14.6 – 23.1  17.8 ± 1.9 | 12.8 ± 5.4 | 11.8 ± 5.0 | 11.9 ± 6.3 | 2.7 | 0.69  [0.55, 0.78] | 2.2 | 0.82  [0.63, 0.91] |
| Overweight  (n = 11) | 12.1 ± 1.4 | 5/6 | 21.0 – 30.7  23.7 ± 2.6 | 15.0 ± 6.4 | 12.8 ± 5.5 | 11.1 ± 5.4 | 3.6 | 0.52  [-0.04, 0.84] | 2.8 | 0.80  [0.15, 0.95] |
| Group comparison (p) | 0.513 | 0.003 | <0.001 | 0.308 | 0.676 | 0.727 | n.a. | n.a. | n.a. | n.a. |

SEM: standard error of measurement; ICC: intraclass correlation coefficient; CI: confidence interval; n.a.: not applicable.
